# e-Learning in Medical Education in Sri Lanka: Survey of Medical Undergraduates and New Graduates

**DOI:** 10.2196/22096

**Published:** 2022-02-10

**Authors:** Keshinie Samarasekara

**Affiliations:** 1 University Medical Unit National Hospital of Sri Lanka Colombo Sri Lanka

**Keywords:** medical education, e-learning, Sri Lanka, medical students

## Abstract

**Background:**

Medical education has undergone drastic changes with the advent of novel technologies that enable e-learning. Medical students are increasingly using e-learning methods, and universities have incorporated them into their curricula.

**Objective:**

This study aimed at delineating the pattern of use of e-learning methods among medical undergraduates and new graduates of the Faculty of Medicine, University of Colombo, and identifying the challenges faced by these students in using e-learning methods.

**Methods:**

A cross-sectional descriptive study was conducted in the Faculty of Medicine, University of Colombo, in April 2020, with the participation of current undergraduates and pre-intern medical graduates, using a self-administered questionnaire that collected data on sociodemographic details, pattern of use of learning methods, and challenges faced using e-learning methods.

**Results:**

There were 778 respondents, with a response rate of 65.1% (778/1195). All the study participants used e-learning resources with varying frequencies, and all of them had at least 1 smart device with access to the internet. Electronic versions of standard textbooks (e-books), nonmedical websites, online lectures, medical websites, and medical phone apps were used by the majority. When comparing the extent of use of different learning methods, it appeared that students preferentially used traditional learning methods. The preference was influenced by the year of study and family income. The 3 most commonly used modalities for learning new study material and revising previously learned content were notes on paper material, textbooks (paper version), and e-books. The majority (98.7% [n=768]) of participants have encountered problems using e-learning resources. The most commonly faced problems were unavailability of free-of-charge access to some e-learning methods, expenses related to internet connection, poor connectivity of mobile internet, distractions while using online resources, and lack of storage space on electronic devices.

**Conclusions:**

There is a high uptake of e-learning methods among Sri Lankan medical students. However, when comparing the extent of use of different learning methods, it appeared that students preferentially used traditional learning methods. A majority of the students have encountered problems when using e-learning methods, and most of these problems were related to poor economic status. Universities should take these factors into consideration when developing curricula in medical education.

## Introduction

With the advent of novel technologies and portable smart devices, medical education has undergone a significant transformation worldwide [1]. In its broadest sense, electronic learning, or e-learning, is the use of internet in education [2]. Students are increasingly using e-learning methods to supplement traditional learning methods such as lectures, textbooks, print journals, and tutorials. There is a wide variety of e-learning methods available for medical education, such as online learning platforms, e-books (electronic versions of standard textbooks), e-journals, online question banks, medical websites, and mobile phone apps. Most educational institutions are incorporating these novel e-learning tools to deliver their curricula [3].

Sri Lanka is a middle-income country with a per capita gross domestic product of US $4102 [4]. Medical education is provided solely via state-sector universities in Sri Lanka. Nine state-sector universities provide undergraduate medical education, and approximately 1200 students graduate from these medical faculties each year. Sri Lankan universities predominantly use traditional teaching and learning methods to deliver medical education.

The Faculty of Medicine, University of Colombo, is the oldest and largest medical school in Sri Lanka, which produces approximately 200 medical graduates each year. A group of academics of the Faculty of Medicine, University of Colombo, established a virtual learning environment for medical undergraduates to supplement traditional learning methods over a decade ago [5].

Studies in other countries have demonstrated that e-learning methods are quite popular among medical students, and that these resources are used for learning new material as well as revising previously learned content [6]. Therefore, it is important to study the pattern of use of e-learning methods and challenges faced by Sri Lankan medical students in order to deliver medical education effectively.

This survey was conducted to identify (1) the pattern of use of e-learning methods among medical undergraduates and new graduates of the Faculty of Medicine, University of Colombo, and (2) the challenges faced by these students in using e-learning methods.

## Methods

### Overview

A cross-sectional descriptive study was conducted in the Faculty of Medicine, University of Colombo, in April 2020. This study was carried out with the participation of current undergraduates and pre-intern medical graduates who have completed their undergraduate degree in 2019 and are awaiting the commencement of internship.

Data were collected using a self-administered questionnaire consisting of 3 sections. The first section was on sociodemographic data.

The second section was designed to identify the pattern of use of learning methods. This section contained questions on the type of personal smart devices and internet facilities used, types of learning resources used, and the extent to which the students used different types of learning resources (both traditional and e-learning methods) for learning new material and for revising previously learned content. The extent of use of learning resources was assessed with a 5-point Likert scale (0=never, 1=rarely, 2=sometimes, 3=often, and 4=always).

The third section was on challenges faced in using e-learning methods. The participants were asked to select the challenges they faced from a list provided and were also given the opportunity to add anything that was not already on the list.

The questionnaire was developed on Google Forms. A separate Google Form was developed to obtain informed written consent and was emailed to all current undergraduates and pre-intern medical graduates. Those who consented were sent the link to fill and submit the questionnaire.

Data were collected anonymously onto a spreadsheet on Google Sheets and analyzed using SPSS, version 25 (IBM Corp). Descriptive statistics were outlined with frequencies, proportions, and percentages, and were summarized using mean with standard deviation. The significance of dichotomous variables was analyzed using chi-square test and those of continuous variables with one-way analysis of variance (ANOVA) test.

### Ethics Approval

Ethics approval for the study was obtained from the Ethics Review Committee of the National Hospital of Sri Lanka (approval number: ERC/NHSL/2020/012).

### Availability of Data and Materials

Data sets supporting the conclusions of this article are included within the article. Additional data at individual student level cannot be provided as per confidentiality agreement.

## Results

There were 778 respondents, with a response rate of 65.1% (778/1195). Of the 778 participants, 450 (57.8%) were female. The highest percentage of the participants (230/778, 29.6%) were from Colombo district, where the commercial capital of the country is located. Approximately one third (263/778, 33.8%) of the participants had a monthly family income less than 50,000 LKR (US $250). A vast majority (710/778, 91.2%) did not have an income of their own and hence were dependent on their parents’ income. The demographic characteristics of the sample are summarized in Table 1.

**Figure 1:**
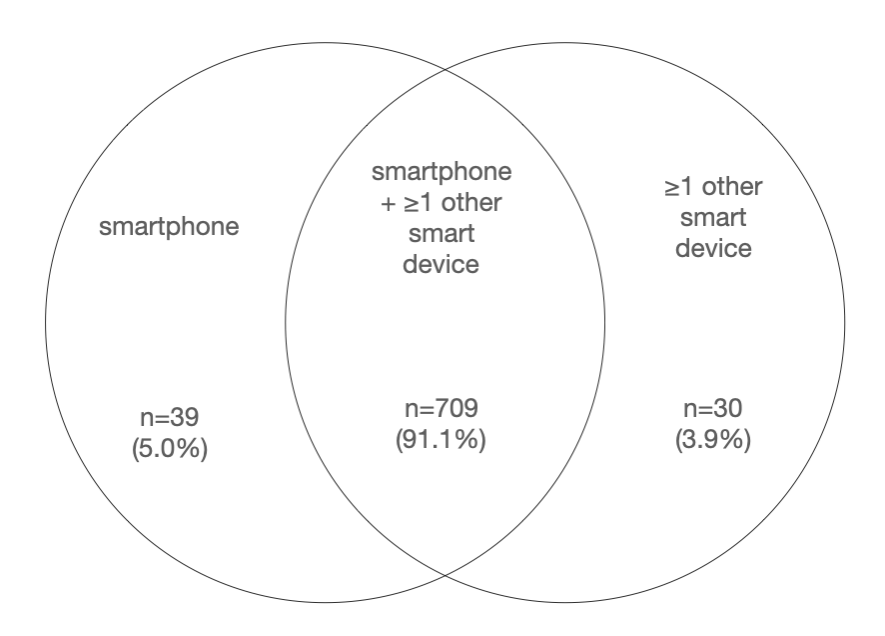
Ownership of smartphones and other smart devices.

A vast majority (748/778, 96.1%) of the study participants owned a smartphone. All those who did not own a smartphone owned some other portable smart-device with internet connectivity such as a tablet/iPad or a laptop. Ninety-one percent of the study participants owned at least one other portable smart device in addition to a smartphone. These data are illustrated in Figure 1.

**Table 1:** Demographic characteristics of the sample.

Characteristics		Values	
**Gender, n (%)**			
	Male		328 (42.2)
	Female		450 (57.8)
Age (years), mean (SD; range)		23.37 (2.19; 19-28)	
**District of residence, n (%)**			
	Within Colombo		230 (29.6)
	Outside Colombo		548 (70.4)
**Family income^a^, n (%)**			
	<50,000		263 (33.8)
	50,001-100,000		245 (31.5)
	100,001-150,000		87 (11.2)
	>150,000		168 (21.6)
	Not answered		15 (1.9)
**Having own income, n (%)**			
	Yes		62 (8.0)
	No		710 (91.2)
	Not answered		6 (0.8)

^a^Family income is in LKR; 200 LKR is approximately US $1.

All participants had access to the internet. A majority (394/778, 50.6%) connected to the internet using both Wi-Fi and cellular data. The rest used either Wi-Fi only (45/778, 5.8%) or cellular data only (339/778, 43.6%).

All of the study participants used e-learning methods. Electronic versions of standard textbooks (e-books), nonmedical websites (eg, Wikipedia), online lectures, medical websites, and medical phone apps were used by the majority of study participants. The percentage of participants using different types of e-learning resources are summarized in Table 2.

A majority (483/778, 62.1%) used e-learning methods for learning new material as well as for revising previously learned content, whereas 205 (26.3%) used it only for learning new material, and 90 (11.6%) used it only for revising previously learned content.

**Figure 2:**
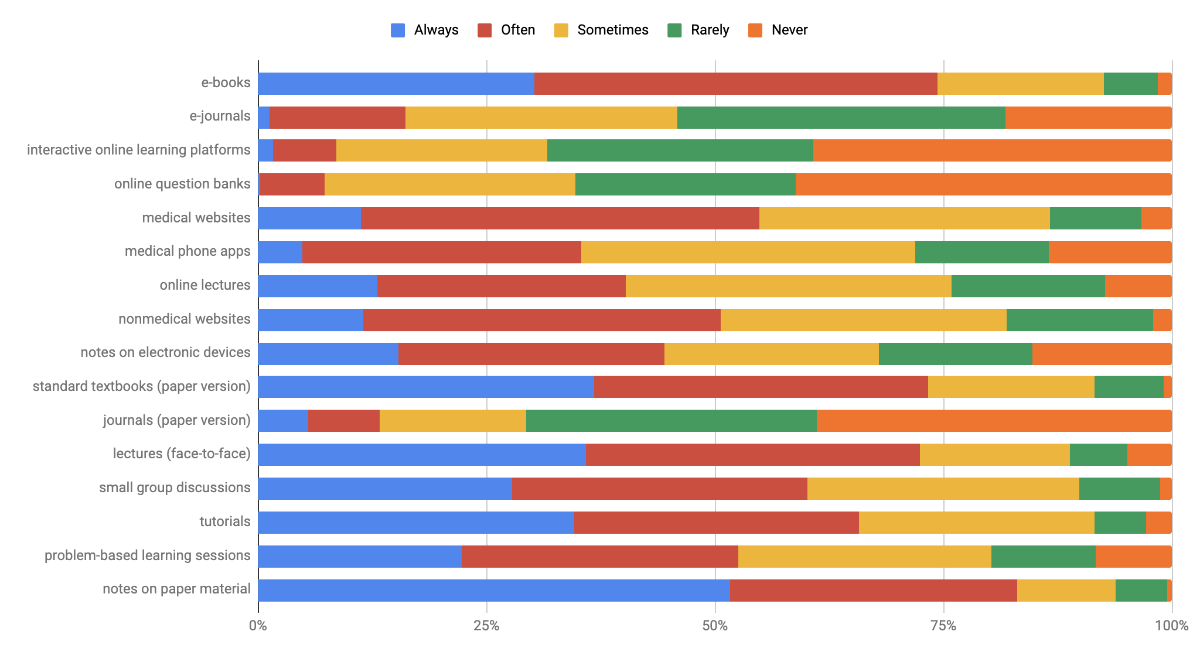
Extent of use of learning methods for learning new material.

**Figure 3:**
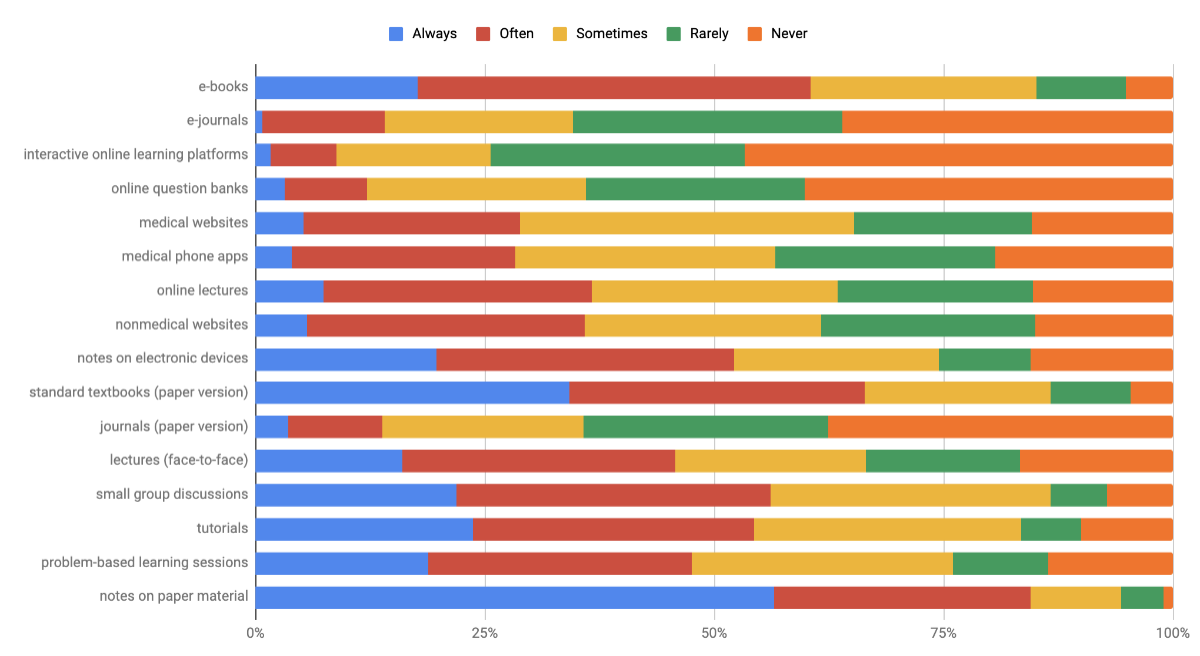
Extent of use of learning methods for revising previously learned content.

The extent of use of different methods (both traditional and e-learning) for learning new material and for revising previously learned content are illustrated in Figure 2 and Figure 3, respectively.

**Table 2:** Percentage of participants using e-learning modalities (n=778).

e-Learning modality	Participants, n (%)
e-Books (electronic versions of standard textbooks)	704 (90.5)
Nonmedical websites (eg, Wikipedia)	528 (67.9)
Online lectures	525 (67.5)
Medical websites	515 (66.2)
Medical phone apps	399 (51.3)
Self-made notes on electronic devices	302 (38.8)
e-Journals	223 (28.7)
Interactive online learning platforms	158 (20.3)
Online question banks	129 (16.6)

The 3 most commonly used modalities for learning new material were notes on paper material, e-books, and textbooks (paper version). The 3 least used modalities were online question banks, interactive online learning platforms, and journals (paper version).

The 3 most commonly used modalities for revising previously learned content were notes on paper material, textbooks (paper version), and e-books. The 3 least used modalities were interactive online learning platforms, online question banks, and e-journals.

A score was assigned for the use of each type of resource according to the extent of use, which is as follows: “Never=0,” “Rarely=1,” “Sometimes=2,” “Often=3,” and “Always=4.” Each participant’s score for using e-learning methods (e-learning score) was calculated by adding the scores for e-books, e-journals, interactive online learning platforms, online question banks, medical websites, medical phone apps, online lectures, nonmedical websites, and self-made notes on electronic devices, and dividing by the number of items. The score for using traditional learning methods (traditional methods score) was calculated in a similar manner by adding the scores for standard textbooks, journals, face-to-face lectures, small group discussions, tutorials, problem-based learning sessions, and notes on paper material, and dividing by the number of items.

The mean “e-learning score” was 1.74 (SD 0.695), and the mean “traditional methods score” was 2.38 (SD 0.759), with a statistically significant difference between the two (*P*<.001).

Chi-square test was used for determining factors associated with the preferred type of learning methods (Table 3).

The preferred type of learning methods was influenced by the year of study and family income (*P*<.001).

One-way ANOVA test was used to determine factors influencing “e-learning score” and “traditional methods score” (Table 4).

The extent of using e-learning methods was influenced by the year of study (*P*<.001), gender (*P*=.003), family income (*P*=.01), and having one’s own income (*P*<.001), whereas the extent of using traditional learning methods was influenced by gender (*P*<.001), district of residence (*P*=.01), and having an own income (*P*=.003).

The problems encountered by students in using e-learning methods and the percentage of participants experiencing each of these problems are summarized in Table 5.

The vast majority (768/778, 98.7%) of the participants have encountered at least 1 problem when using e-learning resources. The challenges faced by the majority include unavailability of free-of-charge access to some e-learning methods (eg, journals), expenses related to internet connection, poor connectivity of mobile internet, distractions while using online resources (eg, notifications from other apps), and lack of storage space on electronic devices.

**Table 3:** Analysis of factors associated with the preferred type of learning methods.

Variable			e-Learning methods preferred, n (%)		Traditional learning methods preferred, n (%)		Chi-square (*P* value)
**Year of study**							55.59 (<.001)
	1st year	34 (4.37)		114 (14.65)			
	2nd year	10 (1.29)		120 (15.42)			
	3rd year	20 (2.57)		164 (21.07)			
	4th year	15 (1.93)		105 (13.49)			
	5th year	55 (7.07)		95 (12.21)			
	Pre-intern	10 (1.29)		30 (3.86)			
**Gender**							1.69 (.19)
	Male	67 (8.61)		255 (32.78)			
	Female	77 (9.89)		373 (47.94)			
**District of residence**							5.13 (.02)
	Within Colombo	31 (3.98)		195 (25.06)			
	Outside Colombo	113 (14.52)		433 (55.66)			
**Family income^a^**							22.27 (<.001)
	<50,000	39 (5.01)		224 (28.79)			
	50,001-100,000	39 (5.01)		206 (26.48)			
	100,001-150,000	16 (2.06)		71 (9.13)			
	>150,000	26 (3.34)		53 (6.81)			
	Not answered	7 (0.89)		8 (1.03)			
**Having own income**							2.02 (.36)
	Yes	14 (1.79)		48 (6.17)			
	No	130 (16.71)		574 (73.78)			
	Not answered	0		6 (0.77)			

^a^Family income is in LKR; 200 LKR is approximately US $1.

**Table 4:** Factors influencing the extent of using e-learning and traditional learning methods.

Variable		e-Learning score, mean (SD)	*P* value		Traditional methods score, mean (SD)		*P* value	
**Year of study**				<.001				.02
	1st year	1.89 (0.71)				2.42 (0.86)		
	2nd year	1.47 (0.76)				2.39 (0.59)		
	3rd year	1.55 (0.72)				2.23 (0.81)		
	4th year	1.73 (0.59)				2.47 (0.64)		
	5th year	1.96 (0.56)				2.41 (0.80)		
	Pre-intern	2.19 (0.42)				2.62 (0.66)		
**Gender**				.003				<.001
	Male	1.66 (0.77)				2.19 (0.76)		
	Female	1.81 (0.63)				2.53 (0.73)		
**District of residence**				.95				.01
	Within Colombo	1.75 (0.75)				2.49 (0.70)		
	Outside Colombo	1.74 (0.67)				2.34 (0.78)		
**Family income^a^**				.01				.04
	<50,000	1.67 (0.67)				2.38 (0.82)		
	50,001-100,000	1.81 (0.66)				2.49 (0.71)		
	100,001-150,000	1.57 (0.77)				2.30 (0.66)		
	>150,000	1.85 (0.70)				2.29 (0.77)		
	Not answered	1.73 (0.89)				2.19 (0.67)		
**Having own income**				<.001				.003
	Yes	2.22 (0.49)				2.70 (0.75)		
	No	1.71 (0.69)				2.36 (0.76)		
	Not answered	1.37 (0.20)				2.45 (0.29)		

^a^Family income is in LKR; 200 LKR is approximately US $1.

**Table 5:** Problems encountered using e-learning methods (n=778).

Problem encountered using e-learning methods	Participants experiencing the problem, n (%)
Unavailability of free-of-charge access to some e-learning methods (eg, journals)	460 (59.1)
Expenses related to internet connection	435 (55.9)
Poor connectivity of mobile internet	426 (54.8)
Distractions while using online resources (eg, notifications from other apps)	409 (52.5)
Lack of storage space on electronic devices	401 (51.5)
Lack of awareness of available free e-learning resources	302 (38.8)
Difficulty in identifying authentic learning material on the internet	285 (36.6)
Poor availability of internet connection	196 (25.2)
Lack of time to use e-learning methods	137 (17.6)
Unwillingness to use technology	102 (13.1)
Poor availability of electronic devices	83 (10.7)
Eye strain	80 (10.3)
Language barrier	75 (9.6)
No problems encountered	10 (1.3)

## Discussion

### Principal Findings

This is the first study in Sri Lanka to identify the pattern of using e-learning resources by medical students and the challenges faced by these students in using e-learning methods.

It showed that all of the study participants used e-learning resources with varying frequencies for learning new content and revising previously learned content, and that all of them had at least 1 smart device with access to the internet.

The most commonly used e-learning modalities were electronic versions of standard textbooks (e-books), nonmedical websites (eg, Wikipedia), online lectures, medical websites, and medical phone apps.

When a score was assigned for use of each type of resource according to the extent of use, the “traditional methods score” was significantly higher than the “e-learning score,” indicating that students preferentially used traditional learning methods. The preferred type of learning methods was influenced by the year of study and family income. A higher proportion of participants in lower-income categories preferred traditional learning methods over e-learning methods. This might be due to the costs associated with mobile devices and internet connectivity.

The extent of using e-learning methods was influenced by the year of study, gender, family income, and having one’s own income. The extent of using traditional learning methods was influenced by gender, district of residence, and having one’s own income. It is interesting to note that some of these factors overlap. Female participants and those with their own income use both e-learning methods and traditional methods more than their respective counterparts.

Most of the challenges encountered in using e-learning resources stem from poor economic status.

Sri Lankan data on the topic of e-learning in medical education are limited. A study carried out on second-year medical students (n=138) of the Faculty of Medicine, University of Colombo, to assess computer literacy and attitudes toward e-learning in 2012 had shown that 93.5% of students owned a computer, and 95% of them had an internet connection [7]. However, the majority of students (65.7%) spent less time on their computer for learning purposes. When comparing these findings with that of this study, it is evident that there is an increase in the available resources as well as using e-learning in medical education in Sri Lanka over the past 8 years.

It is also important to look at studies on e-learning in medical education from other countries for comparison.

A 2017 study carried out on first-year medical students (n=284) of University of Nigeria Teaching Hospital, Enugu State, Nigeria, to assess their readiness for e-learning had shown that 76.1% had access to laptops [8]. It had also shown that these students were ready to move beyond the traditional face-to-face approach, believing that e-learning will improve the quality of their learning.

A 2014 study performed on students (n=270) of Shiraz University of Medical Sciences, Iran, had shown that although the majority (78.5%) of students owned personal computers, only 21.3% used them regularly for learning [9]. Poor connectivity had been the main limiting factor for internet use.

When compared to other middle-income countries, Sri Lankan medical students appear to have better facilities and a better uptake of e-learning resources despite the challenges they face.

In a 2009 study conducted among second-year medical students (n=269) at the School of Medicine and Dentistry at Queen's University Belfast, Ireland, to assess the place for e-learning in clinical skills, the majority (89.2%) of the respondents had their own computer, and 99.6% of them had internet connectivity [10].

A study carried out on penultimate and final year medical students (n=350) of University of Sydney and University of New South Wales, Australia, had shown that, in 2019, despite a general trend toward using e-learning methods, traditional methods such as attending face-to-face lectures remain popular for learning new material [6]. This indicates that, even in more affluent countries, traditional teaching and learning methods still play a major role in medical education.

Medical faculties in Sri Lanka can take the findings of this study into account when developing curricula for their students. Effective e-learning modalities should be used to supplement traditional teaching and learning methods. When using e-learning methods, measures should be taken to minimize difficulties encountered by students. For example, e-learning resources could be developed in such a way that even students with weak internet connections are able to access them. Institutional access for paid online learning resources could be provided to students. A stipend to cover expenses related to internet connectivity and loan facilities to purchase mobile devices and data storage devices could be provided for students with economic difficulties. Moreover, libraries could purchase electronic versions of standard textbooks and provide free access to students.

There are some limitations in this study. This study was conducted in 1 medical faculty, and it might not be possible to generalize the findings to other medical schools in the country. However, similar trends have been observed in studies conducted in other countries, indicating that the trends may not vary greatly in other institutions.

The questionnaire was sent to students via email as a Google Form, which requires a smart device with internet connectivity for access. Therefore, it is possible that those who responded are more likely to use e-learning methods than those who did not.

Further qualitative studies are recommended to gain a deeper understanding and to find measures to overcome challenges faced by medical students in using e-learning methods in Sri Lanka. It is also important to study the factors influencing delivery of medical education via e-learning methods and the challenges faced by educators in preparing e-teaching material.

### Conclusions

This cross-sectional survey from the largest medical faculty of Sri Lanka showed that there is a high uptake of e-learning methods among Sri Lankan medical students. However, when comparing the extent of use of different types of learning methods, it was evident that students preferentially used traditional learning methods. A majority of the students encountered problems when using e-learning methods, and most of these problems were related to poor economic status. Universities should take these factors into consideration when developing curricula in medical education.

## Abbreviations

ANOVA: analysis of variance
